# Editorial: Metabolism of plant natural products -proceedings of annual meeting of CSPB2021

**DOI:** 10.3389/fpls.2023.1120573

**Published:** 2023-02-15

**Authors:** Sheng-Xiong Huang, Xiaoquan Qi, Yong Wang

**Affiliations:** ^1^ State Key Laboratory of Phytochemistry and Plant Resources in West China, and Chinese Academy of Sciences (CAS) Center for Excellence in Molecular Plant Sciences, Kunming Institute of Botany, Chinese Academy of Sciences, Kunming, China; ^2^ Key Laboratory of Plant Molecular Physiology, Institute of Botany, Chinese Academy of Sciences, Beijing, China; ^3^ Chinese Academy of Sciences (CAS)-Key Laboratory of Synthetic Biology, Chinese Academy of Sciences (CAS) Centre for Excellence in Molecular Plant Sciences, Institute of Plant Physiology and Ecology, Chinese Academy of Sciences, Shanghai, China

**Keywords:** metabolism, natural product, bioactive compounds, biosynthesis, metabolomics

Plants, as sessile species, have evolved sophisticated structural and chemical mechanisms to deal with changing environmental conditions. Plants produce a variety of natural products that protect them from pests, diseases, UV-B damage, and other stresses. Terpenoids, phenylpropanoids, and alkaloids are the three major families of plant natural products. Over the last few decades, advances in sequencing technologies and genome editing have boosted our understanding of the biochemistry and genetics of plant natural product biosynthesis. Since natural plant products are a rich source of bioactive compounds for drug discovery, investigating the rational design of high-value products by metabolic engineering or plant breeding would benefit not only plant science but also human life.

This Research Topic aims to present knowledge and progress in plant natural products with a focus on 1) synthesis and function of plant metabolites; 2) plant metabolomics and its applications; 3) biosynthesis and metabolic engineering of plant secondary metabolites; 4) structure and function of plant-derived peptides and toxic proteins; and 5) secondary metabolites-mediated plants-microorganism interactions.

One study, using integrated metabolome and transcriptome analysis, Niu et al. identified a chalcone synthase (CpCHS1) involved in flavonoids and anthocyanidins biosynthesis in tropical fern *Cyclosorus parasiticus*. As a well-characterized enzyme in higher plants, CHS is responsible for catalyzing the sequential condensation of three malonyl-CoA with a *p*-coumaroyl-CoA, generating the first committed precursor naringenin chalcone in flavonoid biosynthetic pathway. Although numerous *CHS* genes have been found in seed plants, few have been identified in ferns. In this study, Niu et al. chose *C*. *parasiticus* fronds at two different stages as materials and found that they contained different amount of flavonoid and anthocyanin metabolites. Therefore, transcriptome-based gene expression analysis was performed to screen candidate genes involved in flavonoids and anthocyanidins biosynthesis. However, by combining *in vitro* enzyme activity assays and protein crystal structure analysis, they finally concluded that CpCHS1 has a highly similar conformation and shares a similar general catalytic mechanism to other plants CHSs. This study provided evidence supporting the conserved evolutionary of flavonoids and anthocyanidins biosynthetic machinery in plants.

A study performed by Xu et al. also focused on flavonoids biosynthesis in plant. They successfully identified a type IV chalcone isomerase, AfCHIL from *Allium fistulosum*, a traditional vegetable rich in flavonoids. Subcellular localization experiments indicated that AfCHIL was localized to the cytoplasm and nucleus. Importantly, the expression pattern of *AfCHIL* transcripts is closely related to the tissue-specific accumulation of anthocyanins in *A*. *fistulosum*. However, instead of catalyzing the formation of naringenin from naringenin chalcone, AfCHIL was found to be responsible for enhancing the activity of CHS through protein interaction. Consequently, in the presence of AfCHIL, the production of naringenin in genetically engineered *Escherichia coli* increased by 39.24%, providing new insight into improving the synthesis efficiency of naringenin through synthetic biology.

In addition to biosynthetic genes, transcription factors also play important roles in regulating plant natural product production. In one study, Zeng et al., identified a WRKY transcription factor, BcWRKY1 in *Baphicacanthus cusia*, a medicinal plant exhibiting remarkable antiviral, antibiosis and anti-inflammatory properties. As a nucleus-localized transcription factor, BcWRKY1 showed transcriptional activation activity. Metabolic profile of *Arabidopsis thaliana* overexpressing BcWRKY1 showed increased abundance of flavonoid- and indole-related metabolites. Consistent with this finding, the expression of biosynthetic genes involved in flavonoid or indole was found to be up-regulated. These results suggested that transcription factor BcWRKY1 might be involved in the metabolic regulation of effective substances in *B*. *cusia*.

Terpenoids are one of the largest groups of plant secondary metabolites. They are usually the main bioactive constituents of essential oils. In the study conducted by Yi et al., 53 *AarbHLH* (basic helix-loop-helix) transcription factor genes were identified from the transcriptome of *Artemisia argyi*, a valuable traditional medicinal plant in Asia. In view of the significant antibacterial, antioxidant, antitumor, analgesic, antiasthmatic, and immunoregulatory activities of the essential oil from leaves of *A*. *argyi*, the authors aimed to investigate the regulation mechanism of *AarbHLH* genes in terpenoid biosynthesis to improve the quality of *A. argyi*. Integrated analysis of the expression profiles of *AarbHLH* genes and the contents of targeted terpenoids, 1,8-cineole and β-caryophyllene in different tissues of *A*. *argyi* led to the identification of 12 *AarbHLH* candidate genes possibly involved in regulation of terpenoid biosynthesis. Moreover, protein–protein interaction networks were constructed to identify the interactions among AarbHLHs and terpenoid biosynthesis enzyme proteins, providing more clues for understanding the potential regulatory mechanism of AarbHLHs in terpenoid metabolism.

Owing to the high chemodiversity of secondary metabolites in plants, their multidimensional applications in human life have been established. In a study performed by Chen et al., they found that the aqueous extract of *A*. *argyi* could inhibit the growth of four harmful weeds to different degrees. UPLC/Q-TOF-MS analysis of the chemical constituents of the aqueous extract identified 13 phenolic compounds and one organic acid, among which caffeic acid (CA) were the most abundant phenolic acid and showed the best allelopathic inhibitory effects. Further transcriptome analysis found that CA inhibited weed growth by downregulating multiple genes involved in gibberellin and phytoalexin biosynthesis and Mitogen-activated protein kinase signaling pathways. In the future, the aqueous extract of *A*. *argyi* or CA could be applied to the development of botanical herbicides. In another study conducted by Rangel et al., nanoemulsion of a butanol-soluble fraction of *Sideroxylon obtusifolium*, a medicinal tree species found in the Restinga and Caatinga areas, was found to possess promising activity against schistosomiasis. The major substances in this extract have been identified as quercetin-3-rhamnosyl-(1-6)-galactoside and hyperoside. This result provided an excellent alternative in controlling and fighting against schistosomiasis.

In summary, these studies reported here demonstrated that integrated metabolome and transcriptome analysis is a promising approach for screening gene candidates involved in biosynthesis of natural products of interest. Moreover, the high chemodiversity of secondary metabolites in plants led to their multidimensional applications in human life.

## Author contributions

All authors listed have made a substantial, direct, and intellectual contribution to the work and approved it for publication.

